# Arthroscopic Humeroplasty in the Treatment of Acute Reverse Hill-Sachs Lesion Associated With Posterior Shoulder Instability

**DOI:** 10.7759/cureus.76528

**Published:** 2024-12-28

**Authors:** Ana Flávia Resende, Margarida Teixeira, Rui Sousa, Zico Gonçalves, Francisco J Agostinho

**Affiliations:** 1 Orthopaedics and Traumatology, Unidade Local de Saúde de Viseu Dão-Lafões, Viseu, PRT; 2 Orthopedics and Traumatology, Unidade Local de Saúde de Viseu Dão-Lafões, Viseu, PRT

**Keywords:** emergency orthopedics, hills sachs lesion, orthopedics and traumatology, posterior shoulder instability, shoulder instability, shoulder reduction techniques (srt)

## Abstract

Reverse Hill-Sachs lesions (RHSL) are common complications associated with posterior shoulder dislocations and represent a significant challenge for preserving joint stability and function. If untreated, these compression fractures of the anteromedial humeral head can compromise the integrity of the joint, predisposing patients to recurrent instability and arthropathy. While various treatment modalities exist, achieving an anatomic reduction of the defect while preserving the articular cartilage remains a desirable outcome, particularly in acute settings.

This case report describes the successful application of a minimally invasive arthroscopy and fluoroscopy-assisted ballon humeroplasty technique to treat an acute RHSL in a 26-year-old male with bilateral posterior shoulder dislocations following trauma. While one shoulder was managed conservatively, the other exhibited recurrent instability due to an engaging RHSL, which led to surgical intervention.

The procedure employed a ballon system to disimpact the humeral defect and subsequent filling with a calcium phosphate bone substitute. The procedure achieved near-anatomic reconstruction of the humeral head without compromising the integrity of the surrounding articular cartilage. At two years of follow-ups, the patient reported no instability or pain, regained full functional capacity, and achieved excellent outcomes, as demonstrated by high shoulder performance scores. This report highlights a minimally invasive technique that appears promising as a safe, effective, and reliable anatomic treatment for acute RHSL while preserving cartilage.

## Introduction

Posterior dislocation of the shoulder is a rare injury, accounting for approximately 2 to 5% of all shoulder dislocations [1,2]. A Reverse Hill-Sachs lesion (RHSL), which is a compression fracture on the anteromedial aspect of the humeral head [3,4], has been shown to occur in up to 30-90% of cases of posterior shoulder dislocations, depending on the degree of instability and the imaging modality used [1,5,6]. The defect size and location of the RHSL are crucial risk factors for re-dislocation and may influence the need for surgical treatment [2]. Additionally, the gamma angle, measured on axial CT cut between the posterior defect edge and bicipital groove, plays an important role in assessing the likelihood of an RHSL being an engaging lesion: the larger the angle, the greater the risk [6,7].

In the event of a failed closed reduction, the percentage of recurrent instability or RHSL is larger than 20%, and in such cases, surgical intervention is indicated [7]. If surgery is deemed necessary, various options may be considered, such as: Arthroscopic reverse remplisage, open modified McLaughlin procedure, disimpaction/fixation, autograft or allograft replacement, rotational osteotomy, or arthroplasty [4,7]. Acute reduction of the humeral head with the preservation of the impacted cartilage has been an option selected for moderate-size RHSL [7]. Over the years, this approach has evolved from a percutaneous method to an arthroscopy-assisted reduction technique [4].

The purpose of this paper is to describe a clinical case of posterior shoulder instability with RHSL, focusing on the use of an arthroscopy and fluoroscopy-assisted disimpaction technique with a balloon. Furthermore, we will discuss the mid-term results of this technique.

## Case presentation

**Figure 1:**
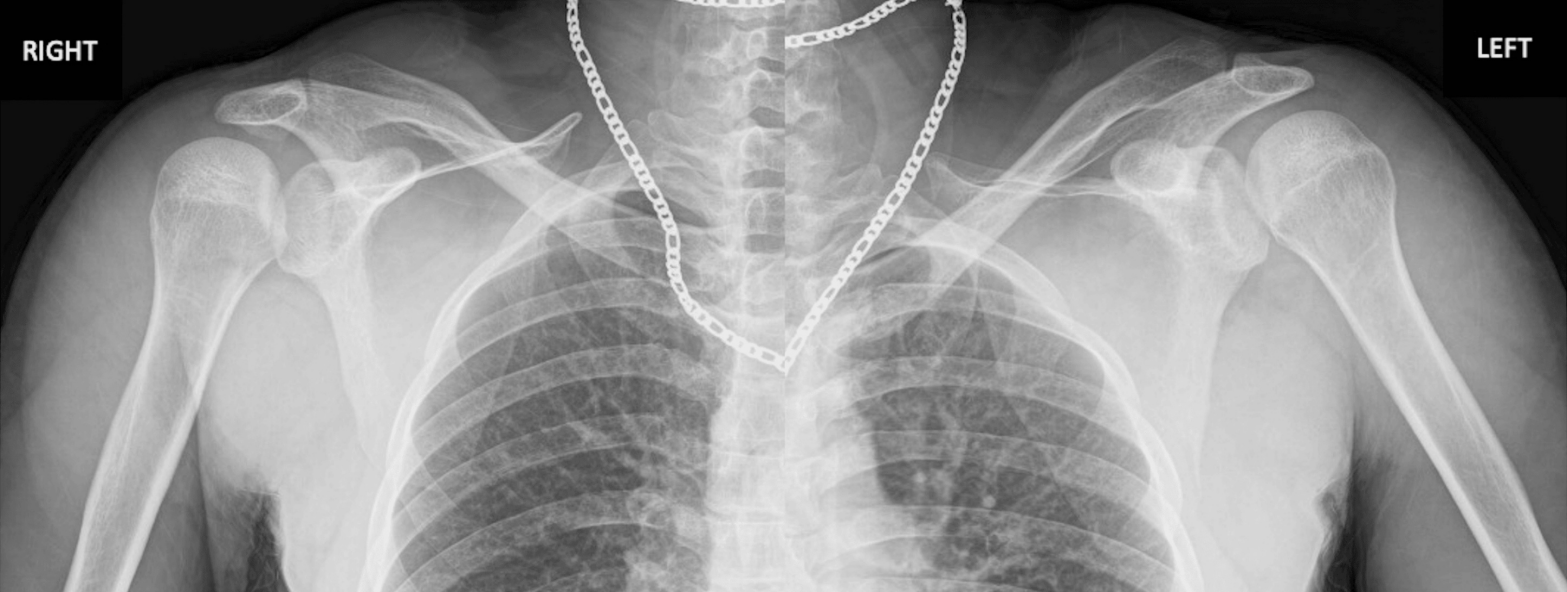
Plain bilateral radiograph of the shoulder showing posterior shoulder dislocation

**Figure 2:**
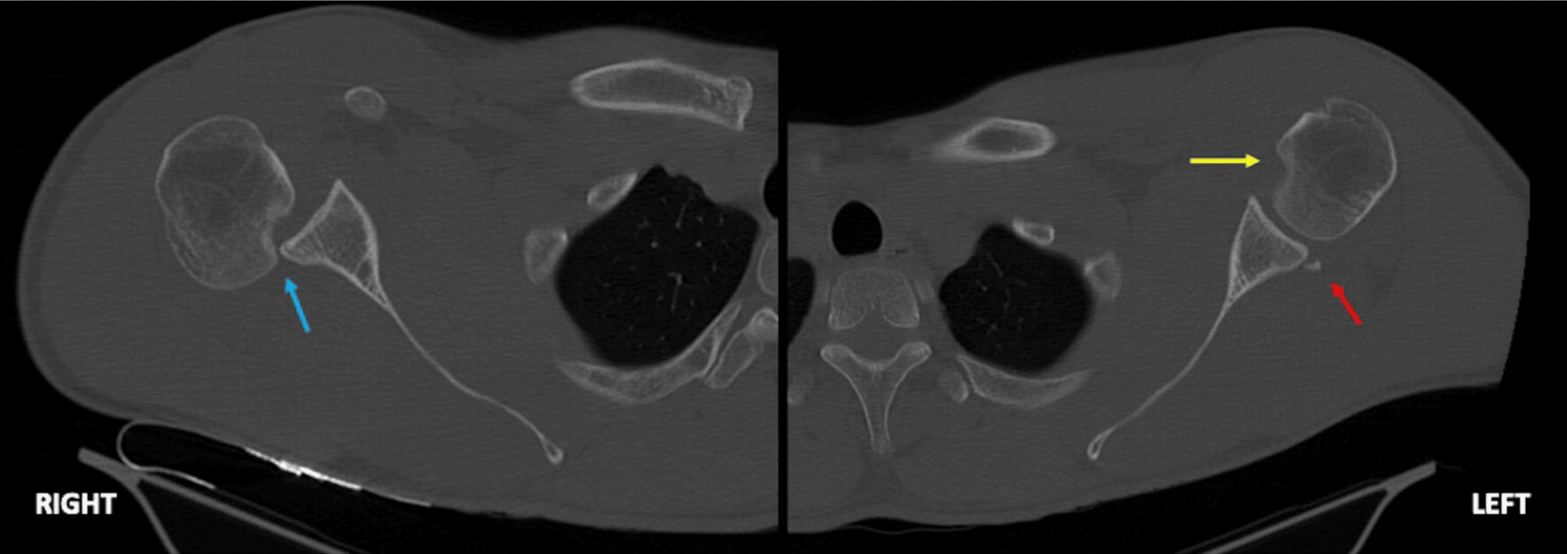
Control bilateral shoulder axial CT scan. The right shoulder scan presented a posterior shoulder dislocation with a Reverse Hill-Sachs lesion (RHSL) - highlighted by the blue arrow - and no lesions on the glenoid were noted. The left shoulder scan revealed a congruent joint with an RHSL and a Reverse Bankart bone lesion- marked by the yellow and red arrow, respectively.

A 26-year-old male presented to the emergency room following a motorcycle accident, reporting direct bilateral anterior shoulder trauma. He complained of bilateral shoulder pain and presented limited external rotation with no evidence of neurovascular deficits. Plain radiographs of the shoulder revealed a posterior bilateral shoulder dislocation (Figure 1). Closed reduction was performed under procedural sedation and analgesia. While the left shoulder was stable after reduction, the right shoulder remained unstable, experiencing several episodes of dislocation in neutral rotation, as shown in the CT scan (Figure 2).

**Figure 3:**
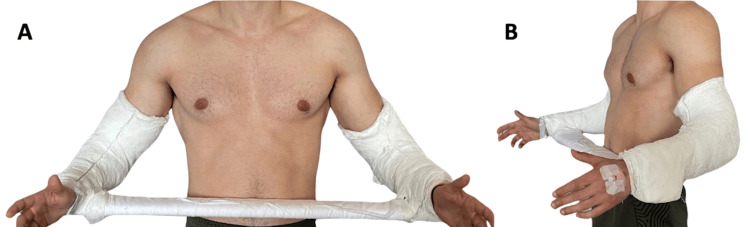
Anterior (A) and lateral (B) views of the patient with bilateral shoulder spica cast, demonstrating immobilization of both shoulders.

**Figure 4:**
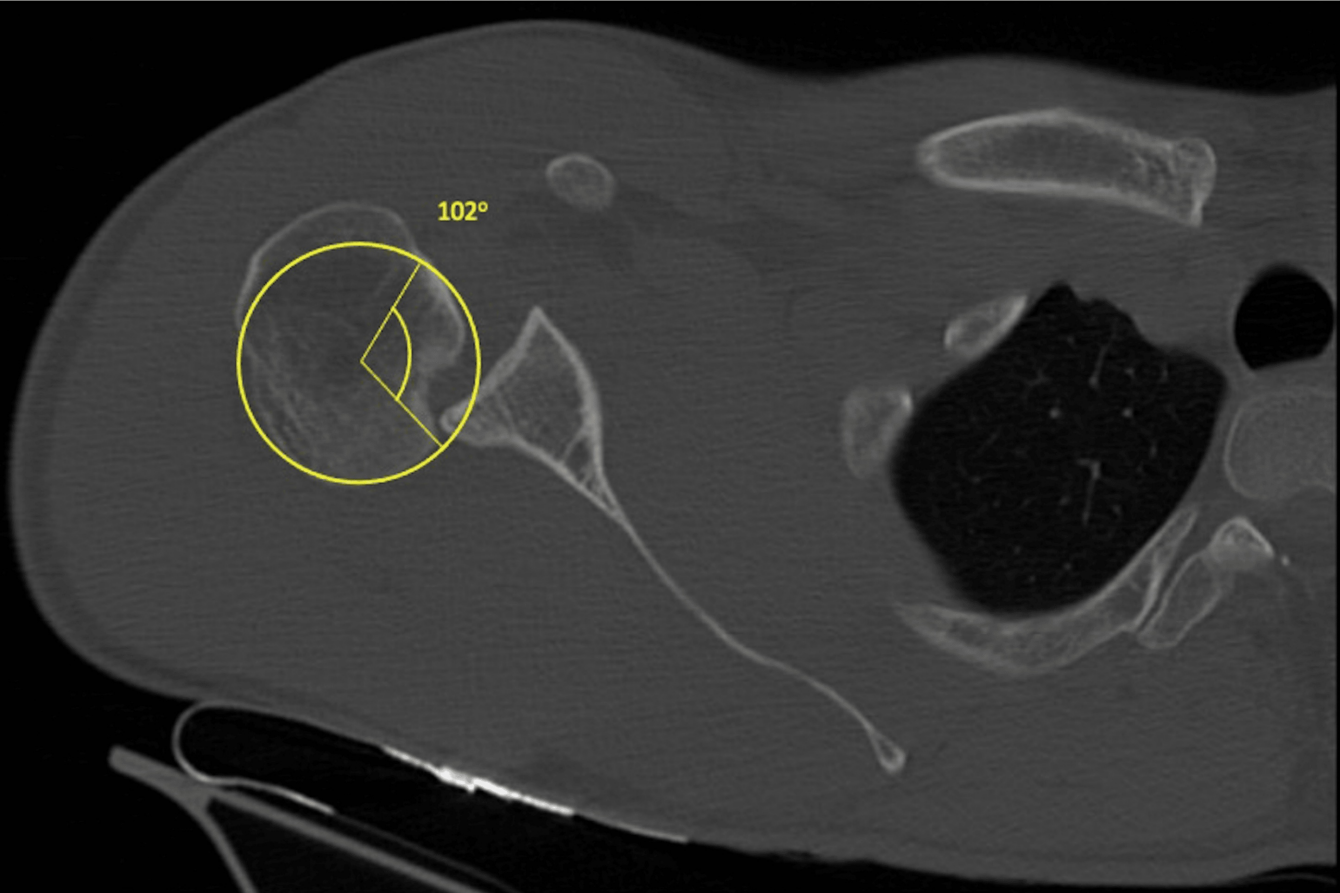
Illustration of the gamma angle in this case's right shoulder The yellow lines display the >90º angle between the posterior defect edge and the bicipital groove, supporting the assumption of an engaging lesion.

Due to the unavailability of conventional slings at our hospital, a bilateral shoulder spica cast was used to maintain shoulder reduction in the abduction and external rotation (Figure 3). Performing a control CT scan after reduction and immobilization was not possible, as the patient was unable to get into the machine at the already referred positioning. A gamma angle >90° was measured in the CT axial scan for the right shoulder (Figure 4), and an engaging lesion was assumed whereby surgery was proposed. Once the opposite side shoulder was stable- despite identifying a reverse bone Bankart lesion- a conservative treatment was established.

The surgical technique followed was based on Ratner's [4] method for arthroscopic reduction and ballon humeroplasty for treating RHSLs. Considering some of the key steps for this technique, we acknowledge that the anterior and posterior labrum revealed no tear; and Kyphon balloon system and calcium phosphate filler (Medtronic) were equally used. However, an intercurrence occurred during this procedure, when the balloon ruptured during the initial inflation, despite the recommended pressure not being exceeded. Afterward, a second balloon was used, but with a more gradual inflation pace, reaching up to 150 mmHg, which proved appropriate.

**Figure 5:**
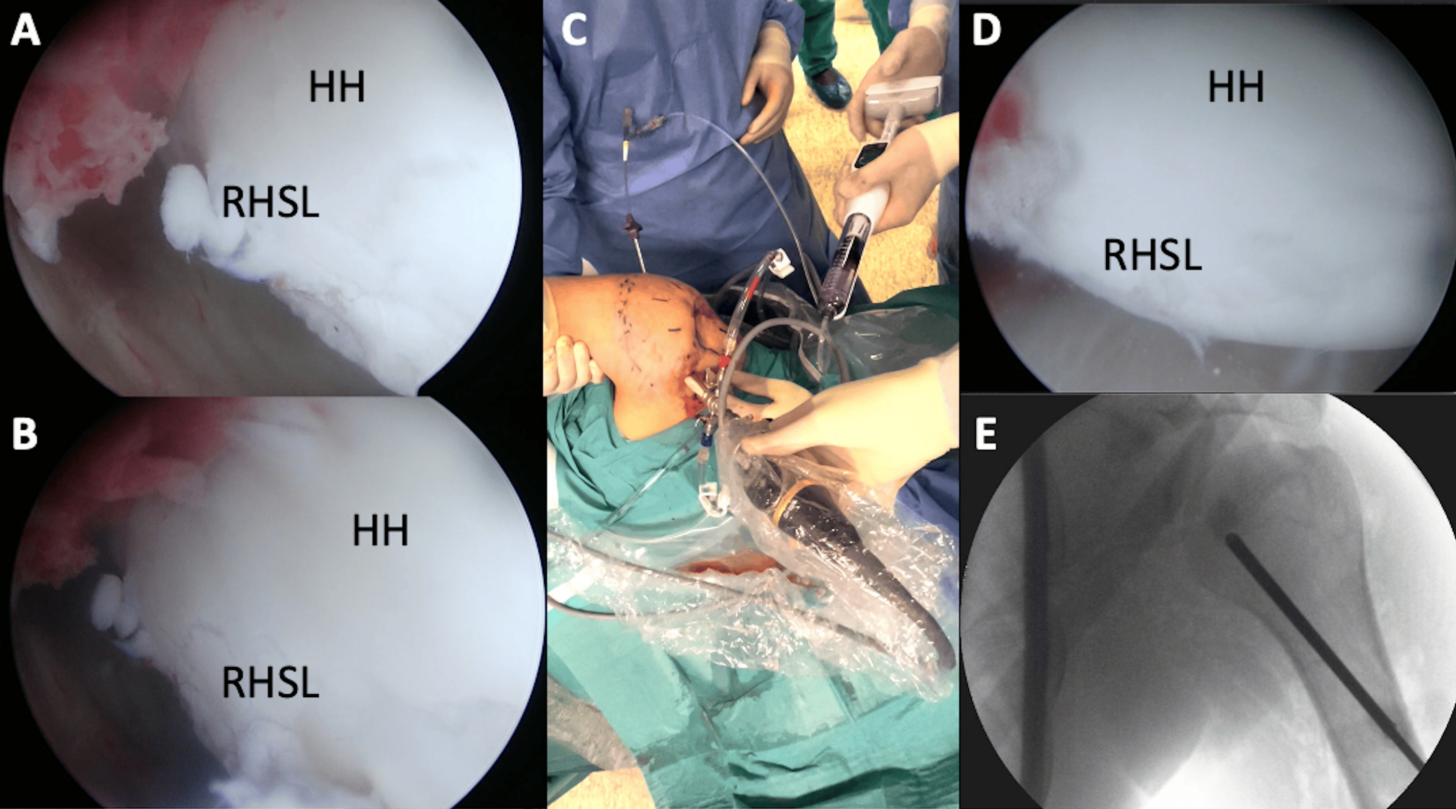
Arthroscopic and fluoroscopic assistance for percutaneous reduction of RHSL This image mosaic demonstrates the arthroscopic approach to RHSL treatment. Panel A shows the arthroscopic view of the RHSL, with a clear depiction of the defect and loss of the humeral head contour. Panel B illustrates the disimpaction of the lesion through balloon insufflation, performed percutaneously using a kyphoplasty system, as shown in panel C. Finally, panel D displays the arthroscopic control, while panel E exhibits the fluoroscopic control, demonstrating the preservation of the lesion reduction after bone filling. HH: Humeral head; RHSL: Reverse-Hill Sachs Lesion

Arthroscopic visualization was employed to confirm that there was no extrusion of the bone filler into the joint. The reduction of the lesion was verified and probed for stability employing arthroscopy, while the dynamic range of motion was tested under arthroscopy and fluoroscopy visualization (Figure 5).

**Figure 6:**
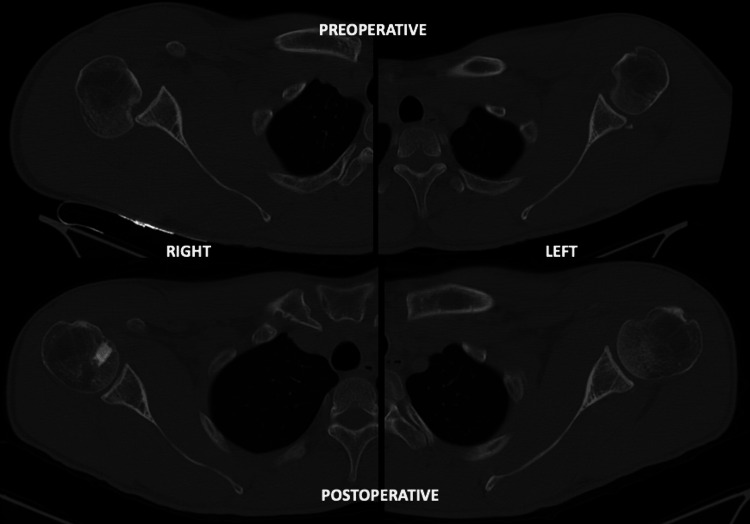
Axial computed tomography comparing preoperative and postoperative RHSL reduction. The image displays axial scans comparing the preoperative and postoperative views of a Reverse Hill-Sachs Lesion (RHSL). The preoperative scan shows the extent of the defect, while the postoperative scan demonstrates a nearly anatomic reduction of the lesion, indicating successful restoration of the humeral head contour and joint congruency.

The patient was placed in an abduction sling, and a CT scan was performed to verify the effective reduction of the lesion and articular congruency (Figure 6).

For postoperative care, a regimen of active motion exercises for the elbow, wrist, and hand, as well as shoulder pendulum exercises, was instituted immediately. The abduction sling was maintained for three weeks, while the limitation of range of motion to neutral internal rotation, 30° of external rotation, and 90° of forward elevation was kept for six weeks. Regarding the left side, the shoulder was not immobilized; however, the rehabilitation protocol was similar to that of the right.

At the two-year postoperative follow-up, the patient reported no complaints, had engaged in his prior leisure activities, and had not experienced any further episodes of dislocation in either shoulder. A constant shoulder score of 96/100 (right side) and 94/100 (left side) was obtained.

## Discussion

Posterior shoulder dislocation associated with RHSL is a significant concern and presents a challenge for shoulder surgeons [8]. The impaction fracture is a well-known risk factor for complications during reduction [5,7], and if left untreated, it may predispose patients to recurrent posterior shoulder instability and the development of arthropathy [3,6].

Dislocations may occur due to direct trauma when the humerus is in flexion, adduction, and internal rotation or through high-energy mechanisms [7]. Particular attention should be given to the abnormal appearance of the shoulder, including internal rotation deformity, posterior fullness, and the prominent appearance of the coracoid process anteriorly. Limitation of external rotation and abduction of the shoulder are key signs during the physical examination [1]. Anteroposterior and lateral radiographs in the plane of the scapula are usually helpful in diagnosing a posterior dislocation and evaluating the presence of a glenoid rim fracture and RHSLs [1,9].

CT imaging is highly valuable for precisely evaluating bony defects in the humerus and glenoid, particularly in surgical planning, assessing ongoing degenerative changes, identifying associated fracture planes, and measuring glenoid retroversion. For assessing the extent of soft tissue injuries, MRI remains the gold standard, with or without applying an intra-articular contrast agent [7,9].

For RHSLs, it is crucial to quantify the defect. Although less well-described than Hill-Sachs lesions (HSLs), the reverse humeral defect can be measured using the "gamma angle." This measurement is taken on the axial cut where the lesion appears widest. A best-fit circle is drawn around the humeral head, and the gamma angle is determined by the intersection of two lines: one connecting the bicipital groove to the center of the best-fit circle and the other connecting the center of the circle to the medial border of the defect. Additionally, if there is glenoid bone loss (measured on the same axial cut by subtracting the glenoid diameter from the best-fit circle's diameter of the glenoid), 2.3° should be added to the gamma angle for each millimeter of posterior bone loss. A gamma angle greater than 90° indicates an engaging lesion that may require surgical treatment [10].

Similar to HSLs, many surgical procedures have been described [8], however, due to the lower incidence of RHSLs, treatment recommendations are largely based on small case series or expert opinions, and thus a consensus has yet to be established [6,7].

Various procedures have been advocated and used in the past to treat RHSL defects, including: "McLaughlin Modified Procedure" - an open approach involving the transposition of the tuberosity and tendon; "Reverse Remplissage" - an arthroscopic procedure in which the middle glenohumeral ligament and subscapularis are reapproximated into the defect; autologous/allograft bone grafting; the "Acromial Bone Block with Pediculated Deltoid Flap" - which has the same mechanical requirements as the coracoid bone block described by Latarjet; and "Rotation Osteotomy of the Proximal Humerus" - where the defect is rotated away from the posterior glenoid. Arthroplasty may also be considered as a treatment option [5,7,11].

These procedures are nonanatomic and less indicated for acute, larger RHSLs [3]. For these lesions, disimpaction and bone grafting have allowed for an anatomic reduction of the humeral head articular surface. Correcting the defect potentially reduces engagement with the glenoid rim and improves stability [7].

Historically, this technique used to be performed via an open approach, creating a trapdoor to access the defect [3]. More recently, a percutaneous technique known as humeroplasty was described by Kazel et al. [12] in a cadaveric study, where they reduced the defect by creating a cortical window and using bone tamping to decompress and elevate the fracture. The resulting void was then packed with allograft. Re et al. [13] and Mehta [14] reported cases of transhumeral head reduction of the Hill-Sachs defect. They used an anterior cruciate ligament (ACL) guide to accurately localize the defect, drill it, and elevate it with bone tamps. Both groups reported good results with no instability at one-year follow-up [13] and six-month follow-up [14].

Sandmann et al. [15] and Stachowicz et al. [8] described the use of a kyphoplasty with subsequent filling of the defect with a bone substitute in cadaver studies. Indirect reduction using an inflation balloon, a technique derived from vertebral kyphoplasty, has more recently been employed to treat limb fractures, including displaced calcaneus fractures and lateral tibial plateau fractures [5]. Their work demonstrated that balloon humeroplasty theoretically reduced acute HSLs to near-anatomical levels. This technique could potentially be used as an adjunct to open or arthroscopic Bankart procedures, allowing surgeons to address engaging HSLs.

Jacquot et al. [5] described four cases of a percutaneous technique in vivo for RHSL and one for HSL [16]. Ratner et al. [12] were the first to describe an arthroscopic-assisted reduction with a balloon.

In this case, we followed Ratner's technique, using arthroscopy and an ACL guide to ensure proper placement of the cortical window in the lateral humerus. Fluoroscopic guidance was also used to confirm the accurate placement of the balloon and cement. With this technique, we were able to achieve a near-anatomic reduction of the RHSL while preserving the articular surface [4,12].

This approach is minimally invasive as it does not alter the rotator cuff muscles, which may facilitate subsequent surgical interventions [4]. Also, this procedure should be performed in an acute setting, though the precise definition of "acute" remains unclear [4].

Regarding the balloon, it should be positioned just beneath the subchondral bone defect to maximize the disimpaction effect. In cases of poor-quality bone, the balloon can cause a blowout of the humeral head, leading to over-reduction. Therefore, a careful increase in balloon pressure and careful selection of patients are important criteria for the success of this technique [8]. In our case, we found the opposite, as the balloon was not strong enough for young, good-quality bone. It is important to understand that balloon kyphoplasty was designed to repair vertebral compression fractures associated with osteoporotic bone [4].

Posteriorly to the execution of the surgery described in this report, additional literature regarding this has been published, including a study by Konrads et al. [2]. These authors highlight the favorable long-term outcomes of joint-preserving surgery involving posterior dislocation with RHSLs in a 10-year follow-up. In their study, the arthroscopic-assisted elevation of the articular surface was reserved for cases where surgery was performed within 14 days of the dislocation, and minimal cartilage damage was present (5 out of 12 patients). This technique did not involve using a balloon or cement to assist the procedure.

More recently, Boulet et al. [3] describe their arthroscopic technique performed in the beach chair position, utilizing bone tamps and a tunnel filled with cement. The technique described in this article appears to be a viable treatment option for RHSLs, with excellent midterm postoperative outcomes. Nevertheless, the strength of these conclusions is limited by the small overall sample sizes reported in the literature [3,4].

## Conclusions

Managing RHSL within the context of posterior shoulder instability is a multifaceted problem. This case report emphasizes using an innovative arthroscopic humeroplasty technique, assisted by fluoroscopy and a balloon, as an effective approach for treating acute RHSL following posterior shoulder dislocation. This minimally invasive method becomes a promising adjunct to surgical interventions in providing a close-to anatomical reconstruction of the humeral head defect without disrupting the articular cartilage.

Although this technique is still a relatively new procedure and only limited case reports have been published so far, the positive outcome in our case-including the restoration of shoulder stability and no recurrent further dislocation-indicates that this could be considered a treatment option in cases of a similar kind. Given the rarity of this injury and the difficulty of establishing standardized treatment protocols, additional studies with larger sample sizes and longer follow-up periods are required to validate this technique's long-term effectiveness and safety.
